# Measures of health-related quality of life and socio-cultural aspects in young patients who after mandible primary reconstruction with free fibula flap

**DOI:** 10.1186/1477-7819-11-250

**Published:** 2013-10-02

**Authors:** Juanfang Zhu, Yan Xiao, Fei Liu, Jing Wang, Wenli Yang, Weihong Xie

**Affiliations:** 1 Department of Stomatolagy, the First Affiliated Hospital of Zhengzhou University, No.1 Jianshe East Road, Zhengzhou, Henan 450052, China

**Keywords:** Free fibula flap, Health-related quality of life, Reconstruction

## Abstract

**Background:**

The reconstruction of mandibular defects after trauma or tumor resection is one of the most challenging problems facing reconstructive surgeons. Although the primary intended outcome of surgery to treat head-and-neck malignancies is still the disease-free survival of the patient, health-related quality of life (HRQOL) is now seen as an essential secondary outcome. This study aims to evaluate HRQOL outcomes in young patients undergoing primary mandible reconstruction with free fibula flap and to collect information about their socio-cultural situation.

**Methods:**

The HRQOL outcomes of 25 young patients after primary mandible reconstruction with free fibula flap for mandible malignancies were assessed using the Medical Outcomes Study-Short Form-36 (MOS SF-36) and University of Washington Quality of Life (UW-QOL) questionnaires 12 months postoperatively.

**Results:**

Using the UW-QOL questionnaire, the best-scoring domain was ‘pain’, whereas ‘chewing’ and ‘anxiety’ were given the lowest scores. Using the MOS SF-36 questionnaire, the best-scoring domain was ‘physical functioning’, while ‘bodily pain’ and ‘general health’ also scored well.

**Conclusions:**

Mandible reconstruction with fibula flap will significantly influence a young patient’s HRQOL. Young patients pay more attention to postoperative facial appearance; this should be considered in surgical planning. The socio-cultural data show a fairly low level of education for the majority of patients.

## Background

The reconstruction of mandibular defects after trauma or tumor resection is one of the most challenging problems facing reconstructive surgeons. Free vascularized fibula and soft tissue transfer is a widely described method of reconstruction of the mandibular defects, particularly when managing oncologic patients [1,2]. A micro-anastomosed fibula flap was first described by Taylor in 1975 [3]. In 1989, Hidalgo reported a significant series of free fibula flaps for mandibular reconstruction [4]. The free vascularized fibula flap provides the longest segment of bone, with 20 to 30 cm available for harvest. This flap can be used to span an angle-to-angle defect. The bone is also of adequate width and height to allow for placement of osseointegrated dental implants [5,6]. Since then, the use of the vascularized free fibula flap as a bone graft has become a cornerstone in the head-and-neck armamentarium. The functional and aesthetic outcomes of the procedure are dictated by the technique used for mandibular reconstruction with bone graft. A number of donor-site morbidities following free fibula flap surgery, although usually minor and transient, have been documented [7]. However, we found that there many patients, especially young patients, were still worried about leg movement after surgery. Although the primary intended outcome of surgery to treat head-and-neck malignancies is still the disease-free survival of the patient, health-related quality of life (HRQOL) is now seen as an essential secondary outcome [8]. It has become an increasingly important outcome measure for patients undergoing treatment for a wide array of illnesses and is a global construct that reflects a patient’s general sense of well-being. It is by definition multi-dimensional and reflective of the patient’s point of view [9].

The aim of our study was to evaluate by questionnaire the HRQOL of young patients who have had mandibular defects after trauma or tumor resection and reconstructions with free fibula flap.

## Methods

### Patients

Our study was granted an exemption in writing by the Ethical Review Board of the First Affiliated Hospital of Zhengzhou University. Patients who had had reconstructive surgery between May 2006 and January 2012 in the Department of Stomatology at the First Affiliated Hospital of Zhengzhou University were eligible. Patients were eligible for inclusion if they had had free fibula flap reconstructive surgery with complete survival at least 12 months previously, were aged between 18 and 45 years, had no previous or synchronous malignancies, had no cognitive impairment. Patients with recurrence of the disease were not excluded.

Thirty-six patients (28 men, 8 women) met the inclusion criteria. Most patients completed the questionnaire when they returned to the hospital for review. The remaining patients received a formal letter explaining the study, an informed consent form, and the Medical Outcomes Study-Short Form-36 (MOS SF-36) and University of Washington Quality of Life (UW-QOL) questionnaires, as well as a questionnaire exploring social and educational status. Patients who did not reply within one month received a reminder. Patients who could not fill in the questionnaires themselves, for example because of dementia or language problems, were excluded from the study.

### Questionnaires and data collection

Although many generic HRQOL measurements have been developed over the past 30 years, the MOS SF-36 [10] and UW-QOL [11] questionnaires are the two most commonly used for cancer patients. The MOS SF-36 questionnaire comprises 36 items that fall into eight health domains: physical functioning, physical role, bodily pain, general health, vitality, social functioning, emotional role, and mental health. Scores can range from 0 (worst) to 100 (best). A Chinese version of the standard MOS SF-36 questionnaire is available and has been validated for a Chinese population [12].

The most recently modified version of the UW-QOL questionnaire was used in this study. This is also available as a Chinese version and has been validated for a Chinese population [13]. The questionnaire covers 15 domains: 12 are disease-specific items (pain, appearance, activity, recreation, swallowing, chewing, speech, shoulder, taste, saliva, mood, and anxiety), and 3 are global questions. Each of the 12 included questions has three to six response options. The domains are scored on a scale ranging from 0 (worst) to 100 (best).

After answering the 15 questions, patients were asked to choose no more than 3 of the 12 disease-specific domains that had been the most important to them in the preceding week.

### Statistical analysis

Data were recorded and analyzed with SPSS 16.0 statistical software. The significance level was set to *P* < 0.05.

## Results

### Patients

Of the 36 questionnaire sent, 25 (69.44%) were completed and returned. In addition, eight replies informed us that the patients had died and two questionnaires were returned, indicating that the patients had changed their address; one patient did not wish to participate. Of the 25 patients who completed questionnaires, there were 20 men and 5 women with a median age of 35.5 (range 18 to 45); the postoperative follow-up period ranged from 12 months to 6 years. Fourteen patients had received treatment between 1 and 3 years previously and the remaining 11 had been treated more than 3 years previously. There had been seven (28.0%) tumors in the body and angle of the mandible, ten (40.0%) that occupied the body-angle ramus of the mandible, and eight (32.0%) in the body of the mandible. All patients had had mandible squamous cell carcinoma.

### Quality of life

#### UW-QOL

The scores for 12 disease-specific domains and the importance of each domain are shown in Table 1. Topping the list of ‘good’-scoring domains were ‘pain’ and ‘shoulder’. 36.0% of patients scored ≥80% in the ‘pain’ domain, with a main score of 76.88 points. The worst scoring domains are recreation, swallowing, and chewing. All patients were dissatisfied with saliva and taste. The most important domain, over the previous week, was ‘appearance’, and this was followed by ‘chewing’ and ‘anxiety’. The ‘pain’ and ‘shoulder’ domains were considered least important.

**Table 1:** Means of scores of items and scales of UW-QOL questionnaire

	**Mean**	**SD**	**Median**	**Range**	***G*ood *score %***^**a**^	***Importance of domains, %***^**a**^	***Rank order***
Pain	76.88	6.87	77.00	65 to 89	*36.0%*	0*%*	9
Appearance	72.08	7.09	73.00	60 to 85	*20.0%*	*72.0%*	1
Activity	61.96	8.16	61.00	50 to 80	*4.0%*	20.0%	6
Recreation	59.52	9.61	61.00	40 to 78	0*%*	12.0%	8
Swallowing	49.80	15.52	43.00	20 to 77	0*%*	*16.0%*	7
Chewing	35.40	14.33	34.00	10 to 70	0*%*	*56.0%*	2
Speech	58.76	11.94	58.00	40 to 84	*8.0%*	*28.0%*	4
Shoulder	76.68	7.71	78.00	60 to 87	*36.0%*	0*%*	9
Taste	74.64	6.45	74.00	60 to 87	*24.0%*	*20.0%*	6
Saliva	50.24	10.83	50.00	40 to 82	*8.00%*	*24.0%*	5
Mood	65.52	14.42	74.00	40 to 85	*12.0%*	0*%*	9
Anxiety	42.88	13.18	45.00	20 to 75	0*%*	52.0%	3

^a^Good score %, percentage scoring ≥80 points; Importance of domains, percentage of patients who considered that domain important (up to three domains per patient).

#### MOS SF-36

The distributions of MOS SF-36 domain scores are shown in Table 2. The best-scoring domains for the complete collective in the MOS SF-36 were ‘physical functioning’ (77.32 points), ‘bodily pain’ (74.56 points), and for ‘general health’ (72.56 points). A total of 32.00% of patients rated ‘physical functioning’ ≥80 points. The lowest scores were for ‘mental health’ and ‘vitality’; the majority of patients said that they worried about their operated limb and reduced vitality.

**Table 2:** Means of scores of items and scales of SF-36 questionnaire

	**Mean**	**SD**	**Median**	**Range**	***Good score %***^**a**^
Physical functioning	77.32	8.55	76.00	60 to 89	32.00%
Role physical	66.96	12.94	71.00	40 to 79	0*%*
Bodily pain	74.56	6.95	74.00	60 to 86	28%
General health	72.56	8.77	75.00	50 to 85	20*%*
Vitality	56.12	9.51	56.00	40 to75	0*%*
Social functioning	66.96	10.38	66.00	50 to 85	16%
Role emotion	66.32	7.84	65.00	54 to 85	8.0%
Mental health	52.56	15.76	54.00	20 to 76	0*%*

^a^Good score %, percentage scoring ≥80.

More than 60% patients had had little education. Three (12.0%) patients did not complete primary education. Thirteen patients (52.0%) had completed an elementary school education and only four patients (16.0%) had reached university graduation. Five patients (20.0%) had graduated from a senior middle school. Two patients could not read or write, and needed help to complete the questionnaire. Only six patients (24.00%) had a stable income, social security and medical insurance; the rest needed funding from their families. 16 patients were married, 9 patients were unmarried and lived with their parents.

## Discussion

The mandible plays a major role in airway protection and support of the tongue, lower dentition, and the muscles of the floor of the mouth, permitting mastication, articulation, deglutition, and respiration. It also defines the contour of the lower third of the face. Interruption of mandibular continuity, therefore, produces both a cosmetic and functional deformity [14]. Deciding on mandible resection and vascularized fibula reconstruction is still difficult for both patients and clinicians. Difficulties in operation, the health of the patient, damages to the donor site, and economic burdens are taken into account.

The expectation of the clinical outcome of reconstruction is regarded to be the most important factor in the decision, while HRQOL measurement provides information about perceptions of patients [15]. Measuring HRQOL is an integral part in assessing the outcome of treatment for patients with oral cancer. As the oral cavity is responsible for many different functions, such as chewing food, swallowing, saliva production, speech, and breathing, and not least for interpersonal contacts, such as kissing, a functional deficit leads to obvious changes to patients’ quality of life [8]. The relatively large number of questionnaires specific for diseases of the oral cavity reflects that there is no ‘gold standard’. Our study used the MOS SF-36 and UW-QOL questionnaires. So far, there have been no data regarding the HRQOL of this specific patient population, which is usually merged with groups of young patient undergoing primary mandible reconstruction. We conducted this cross-sectional study to determine the postoperative HRQOL of these patients and the possible relationship of medical and demographic factors with HRQOL.

Generic questionnaires are usually used to assess general well-being, and pay less attention to a specific disease. The MOS SF-36 questionnaire was used as a generic questionnaire in our study, because it is the most widely evaluated generic patient-assessed health-outcome measure [12] and has been used in head-and-neck cancer patients previously. In our study, the ‘vitality’ components (56.12 ± 9.51) and ‘mental health’ components (52.56 ± 15.76) received significantly low scores; many patients were worried about the movement of the operated limb. Young patients, in particular, are more active, and worry about being unable to carry out normal activities in work and daily life. It is not convenient that the leg should contribute a large psychological burden. The ‘physical functioning’ (77.32 ± 8.55) and ‘bodily pain’ (74.56 ± 6.95) components scored well. Our studies show very few patients scoring ≥80% in all domains; this reveals that oral cancer surgery does seem to have an overall effect on oral health. Patients believe that surgery has considerably damaged their oral function.

The oral-specific questionnaire was able to better demonstrate the changes in quality of life due to surgery. Many scholars have chosen to use the UW-QOL questionnaire [13]; this measure was chosen as the head-and-neck-specific questionnaire because it is short and easy for patients to complete themselves, thus making it ideal in a busy outpatient setting. A remarkable finding was that the highest score given to any of the UW-QOL subscales in this study was given to the ‘pain’ domain. The average score was 76.88 ± 6.87, which indicated a great improvement in the pain domain. Besides the ‘pain’ domain, patients also rated the ‘appearance’ (72.08 ± 7.09) domain highly. This may be because reconstruction using free fibula flaps has provided surgeons with the opportunity to address the aesthetic and functional reconstruction of mandibular defects more carefully, using the wide variety of bone and soft tissue available. The reconstruction of the mandible retained the contour of the lower third of the face, thus maintaining the patient’s facial appearance. We found that the ‘anxiety’ domain (42.88 ± 13.18) received a low score. In the follow-up visit, many young patients said they worried that the operated limb would affect their normal movement. This led to the young people reducing their activity, which led to a reduction in their exchanges with their peers; this may lead to a greater psychological burden in younger patients.

Hsing *et al*. [16], in their study on importance-rating using the UW-QOL questionnaire in patients treated by primary surgery for oral cancer, found that patients tended to rate speech, chewing, and swallowing as more important than the other UW-QOL domains. Our study found different results: appearance, chewing, and anxiety were felt to be most important. This may be because the patients in this study are young people, who set great store by appearance.

We compared the patients’ donor and un-operated (control) legs with regard to a range of motion of ankle inversion and eversion and normal leg movement. All patients indicated that there was no significant difference between the two legs after 12 months. This suggests that the free fibula flap operation has little effect on patients’ donor leg movement. However, many patients were worried that the donor leg would be more prone to injury, and this greatly limited the patients’ movements. This result was similar to that of Farhadi *et al*. [17]; their study showed that the true ankle range of motion for dorsiflexion and plantar flexion was 67.1° for the donor side and 63.6° for the contralateral healthy side (*P* > 0.05).

In our study, discrimination sensitivity differed in 10 out of 25 patients (40.0%) for operated and non-operated limbs. Three of the 25 patients (12.0%) showed sensitivity to pain on pricking, and seven out of 25 patients (28%) showed tactile sensitivity. It is noteworthy that discriminative, tactile, and pain sensitivity were well conserved in the operated limb, which might account for the patients’ being less bothered by their difficulty in walking, which was therefore not their prime complaint. This is similar to the results of Bartaire *et al.*[18].

The socio-cultural data collected highlighted the low education level of our patients. Three (12.0%) patients did not complete primary education. Thirteen patients (52.0%) had completed an elementary school education and only four patients (16.0%) had reached university graduation. Five patients (20.0%) had graduated from a senior middle school. More than half of the patients did not have a steady job, relying on manual labor to maintain their livelihood before their operations. Therefore, they relied on the help of their close family and other relatives to meet their living costs after surgery. It is noteworthy that in our study, more than 60% patients had had little education, and only six patients (24.0%) had a stable income, social security, and medical insurance. Most of these patients had a very poor standard of living, and did not receive timely treatment in the early stages of the disease. In some studies [19], people who were unemployed had a significantly higher risk of cancer (odds ratio = 2.27) than those with high levels of educational attainment. A high risk of head-and-neck cancer was consistently associated with poor socioeconomic circumstances, and there were strong links for specific components.

More than 60% of patients are treated with either chemotherapy or radiotherapy. Previous studies have shown that with adjuvant radiotherapy, weight, salivary function, and physical function were significantly reduced and that swallowing, coughing, and dry mouth symptoms increased [13,20], compared with operation alone. Our work revealed a similar finding: 68% (17/25) patients in our study received postoperative radiotherapy or chemotherapy.

There were several limitations of this study. First, the sample size was small and may not have had sufficient power to find more valuable results. Second, this study only described oral cancer of the study population at one point in time, and thus could not fully assess the young patients’ HRQOL over the whole period of post operation.

## Conclusions

Mandible reconstruction with fibula flap would have significantly influenced young patients’ quality of life, especially young patient’s anxiety and mental health. Young patients pay more attention to postoperative facial appearance; this should be considered in surgical planning. The socio-cultural data showed a rather low education level for the majority of the patients.

## Competing interests

The authors declare that they have no competing interests.

## Authors’ contributions

JZ, YX, FL, and WY participated in the clinical management of the patient and wrote the manuscript. WX and JZ were involved in the final editing. All authors read and approved the final manuscript.
